# The Adverse Effect of Selective Cyclooxygenase-2 Inhibitor on Random Skin Flap Survival in Rats

**DOI:** 10.1371/journal.pone.0082802

**Published:** 2013-12-06

**Authors:** Haiyong Ren, Dingsheng Lin, Zhenyu Mou, Pu Dong

**Affiliations:** Department of Hand and Plastic Surgery, The Second Affiliated Hospital of Wenzhou Medical College, The Second Clinical Medical College of Wenzhou Medical College, Wenzhou, China; University of Catania, Italy

## Abstract

**Background:**

Cyclooxygenase-2(COX-2) inhibitors provide desired analgesic effects after injury or surgery, but evidences suggested they also attenuate wound healing. The study is to investigate the effect of COX-2 inhibitor on random skin flap survival.

**Methods:**

The McFarlane flap model was established in 40 rats and evaluated within two groups, each group gave the same volume of Parecoxib and saline injection for 7 days. The necrotic area of the flap was measured, the specimens of the flap were stained with haematoxylin-eosin(HE) for histologic analysis. Immunohistochemical staining was performed to analyse the level of VEGF and COX-2 .

**Results:**

7 days after operation, the flap necrotic area ratio in study group (66.65±2.81)% was significantly enlarged than that of the control group(48.81±2.33)%(P <0.01). Histological analysis demonstrated angiogenesis with mean vessel density per mm^2^ being lower in study group (15.4±4.4) than in control group (27.2±4.1) (P <0.05). To evaluate the expression of COX-2 and VEGF protein in the intermediate area II in the two groups by immunohistochemistry test .The expression of COX-2 in study group was (1022.45±153.1), and in control group was (2638.05±132.2) (P <0.01). The expression of VEGF in the study and control groups were (2779.45±472.0) vs (4938.05±123.6)(P <0.01).In the COX-2 inhibitor group, the expressions of COX-2 and VEGF protein were remarkably down-regulated as compared with the control group.

**Conclusion:**

Selective COX-2 inhibitor had adverse effect on random skin flap survival. Suppression of neovascularization induced by low level of VEGF was supposed to be the biological mechanism.

## Introduction

For many years, NSAIDs have administered parenterally for the treatment of pain and inflammation associated with acute tissue damage due to injury or surgery. However, the efficacy of traditional non-selective NSAIDs is limited by side effects associated with gastrointestinal ulceration, renal dysfunction, and bleeding caused by platelet inhibition [1].

The selective COX-2 inhibitors, which mediates inflammatory prostaglandin synthesis by inhibiting the inducible COX-2 isoform without suppression of constitutive COX-1, were thought to exert analgesic and anti-inflammatory effects without causing serious adverse effects [2–4]. Thus in recent years, selective inhibition of the COX-2 enzyme was extensively applied in clinical practice for the treatment of pain and inflammatory conditions [5].

However, increasing evidences demonstrating that selective COX-2 inhibitors have the risks to cause greater potential for heart attacks [6–8], strokes and other cardiovascular problems [9,10]. Studies suggested that administration of selective COX-2 inhibitors soon after injury, while providing desired analgesic effects, may attenuate wound healing in many tissues [11] and are associated with a significantly higher incidence of wound infections [12].

Random skin flap is common for repairing wound and reconstructing the function. It can be used throughout the hand and fingers. such as Z-plasty, Limberg flap, Rotational flap [13]. This study was undertaken to investigate the effects of selective COX-2 inhibitor (Parecoxib) on survival of random pattern skin flaps and further explore the mechanism.

## Materials and Methods

### Ethics Statement

This study utilized experiments using rats.This study was carried out in strict accordance with the recommendations in the Guide for the Care and Use of Laboratory Animals of the National Institutes of Health. The protocol was approved by the Committee on the Ethics of Animal Experiments of Wenzhou Medical College(wydw2012-0079). All surgery was performed under Chloral hydrate anesthesia, animals were removed from the study and euthanized by an overdose of Chloral hydrate, and all efforts were made to minimize suffering. The study did not involve human experiments.

### Animals and Materials

Male Sprague-Dawley (SD) rats (250-300 g) were obtained from Wenzhou Medical college (SCXK(zhe) 2005-0019). Parecoxib Sodium for Injection (Pfizer)was obtained from commercially available sources. Anti-VEGF-A polyclonal antibody (pAb), anti-COX-2 pAb and β-Actin pAb were obtained from Bioworld (Nanjing, China). The goat anti-rabbit IgG-R(Santa Cruz Biotech) was obtained as secondary antibodies.

### Flap Model and Experimental Design

The rats were anesthetized using 10% Chloral hydrate (3 ml/kg) by intraperitoneal injections. Dorsal skin was shaved and rats were put to the prone position with their limbs secured by adhesive tape. Then the skin was disinfected with povidone iodine (PI) solution , and all surgical procedures were performed under sterile conditions.

Random dorsal skin flaps were elevated using the model initially described by McFarlane [14] and later modified by Rinsch et al [15]. We outlined caudally-based, 3×9cm large skin/panniculus carnosus flaps on the back of the rats and systematically sectioned both sacral arteries. The flap was completely separated from the underlying fascia up to its base and then immediately sutured back to the donor bed using 4-0 silk on a swedged-on cutting needle. For analysis, the flap area was divided into three distinct zones of equal size reflecting the clinical aspect of complete flap survival in the proximal area I, a mixed pattern of flap survival and partial necrosis in the intermediate area II, and full thickness necrosis in the distal area III [16] .

All rats were housed individually in standard experimental cages, in an environmentally controlled room with regards to temperature and light–dark cycle and were fed standard rat chow and water ad libitum. In case of the rats Self-mutilation [17], each rat was given a neck collar.

### Administration of the drug

Parecoxib Sodium for Injection was dissolved with isotonic sodium chloride. The rats were randomly divided into two groups. The selective COX-2 inhibitor group(n=20) received Parecoxib 10 mg/kg/twice dose a day during 7 days (totally: 20 mg/kg/day), and the saline group (n=20) only received the same amount of isotonic sodium chloride during the experiment. The first drug solution was administered to the animals 2h after the surgical procedure and the drug administered to the animals through intramuscular injections. Seven days later, all animals were euthanized using an overdose of Chloral hydrate. Flaps were excised, photographed and measured. All specimens were put into formalin 10% and were sent for histological analysis and immunohistochemical analysis.

### General Observation and Percentage of Necrotic Area

On the seventh postoperative day, the flap area was photographed and compared with that recorded on the first day of the experiment. Flap necrosis was defined by dark color and eschar formation. The photographs were captured by the computer software Image-Plo Plus v6.0. Mean flap necrotic areas were then assessed for all groups. All the results were represented as mean and standard deviation.

### Histology

After the rats were sacrificed, flap tissues from three portions of all animals were biopsied for histology assessments. Each specimen (1 cm × 1 cm)was fixed in 10% paraformaldehyde for 24 hours, embedded in paraffin, sectioned to 4-µm slices, and prepared for hematoxylin and eosin (H&E) staining. Also, we observed tissue conditions as the thickness of granulation tissue, tissue edema, and neutrophil infiltration under a light microscope (100 × magnification) and calculated the microvessel number of per-unit area (/mm^2^) as indicators of the microvascular density (MVD) [18].

### Immunohistochemistry for COX-2 and VEGF Evaluation

Sections (5 mm) of the paraffin-embedded pedicles were mounted on gelatin-coated glass slides and immunohistochemical staining was carried out for COX-2 and VEGF using the streptavidin-peroxidase method. With the BX51 optical microscope (Olympus Corporation, Tokyo, Japan), we searched for the positive expression of VEGF and COX-2 intensive regions under low magnification, then randomly selected five horizons in each slice under × 400 magnification using the DP2- TWAIN image-acquisition system (Olympus Corporation). We saved the images into Image-Plo Plus v6.0 software (Media Cybernetics, Rockville, Maryland) and detected the integral absorbance (IA) value as an indicator of COX-2 and VEGF expression.

### Western Blot Assay for COX-2 and VEGF

For western blot assay, total cellular protein was extracted from rat tissues (II area) by using a RIPA lysis buffer (Beyotime, Jiangsu, China) containing 50 mM Tris-HCl (pH 7.4), 150 mM NaCl, 1% Triton X-100, 1% sodium deoxycholate, 0.1% SDS, 1 mM EDTA, 1 mg/ml leupeptin, 2.5 mM sodium orthovanadate and 1 mg/ ml aprotinin. The homogenized tissue samples were homogenized and centrifuged at 12,000g for 10 min,at 4°C and the protein concentration of the supernatant was measured by using commercially available Bradford reagent. The proteins were separated by the SDS–PAGE and transferred to polivinyledene fluoride (PVDF) membranes, which were incubated with primary antibodies against VEGF，COX-2（1:1000) and β-actin (1:500) and probed with the respective secondary antibodies. Then the bands were detected with ECL plus reagent (Invitrogen) by the system of enhanced chemiluminescence detection (PerkinElmer, Waltham, MA). At last, the intensity of these bands was quantified by the software of AlphaEaseFC 4.0, and presented in result section as compared with β-actin. This experiment was repeated three times.

### Statistical Analysis

The results were expressed as mean ±SD. All data were analyzed using SPSS software 19.0. Statistical significance was accepted at p<0.05. The degree of necrotic change, as well as histologic and immunohistochemistry results were compared using the Mann-Whitney test. Western blot analysis was compared using Student's t-test.

## Results

### General Observation

**Figure 1:**
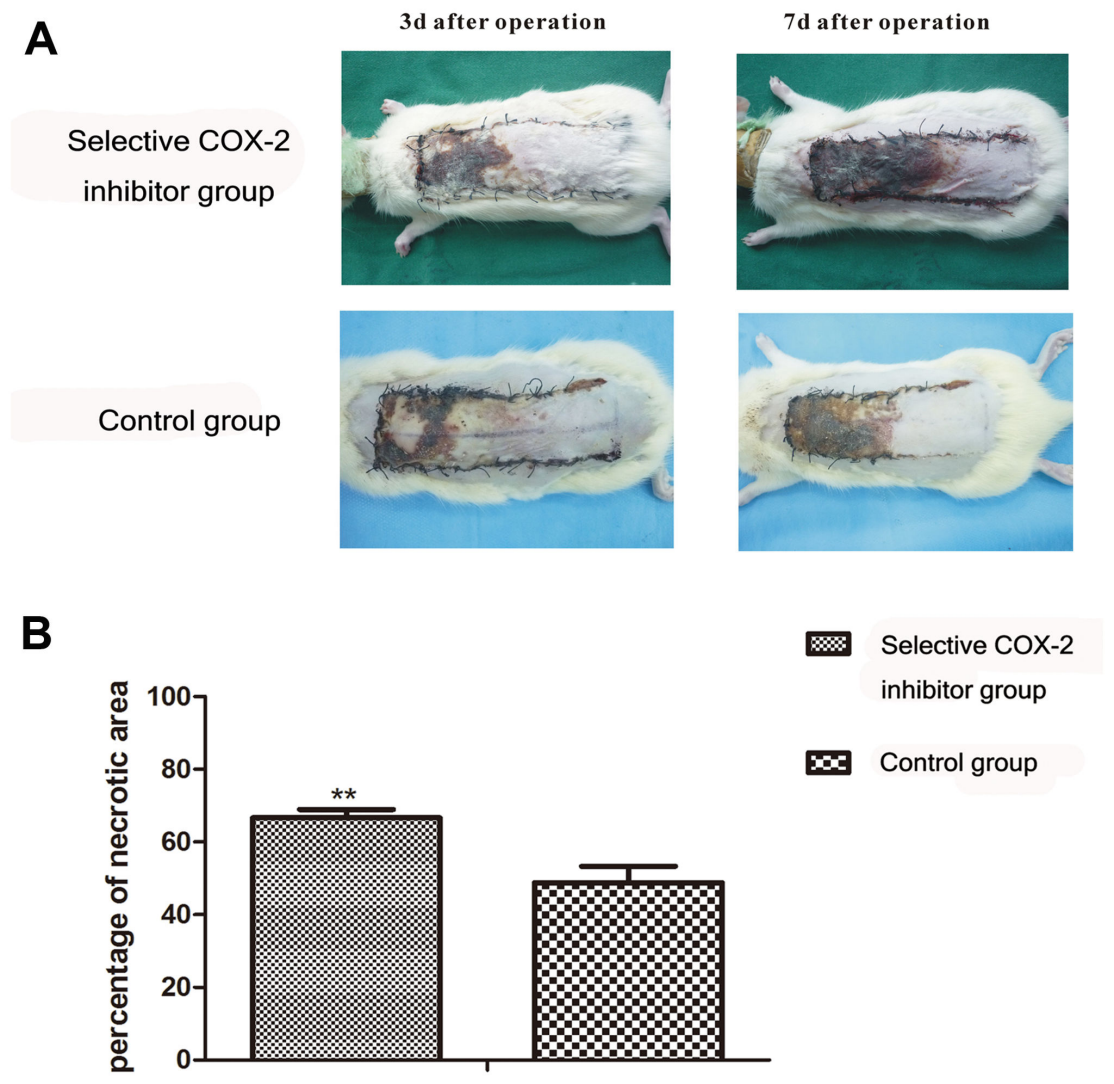
Comparison of the flap necrotic area between the two groups. **A**. Necrosis status of flaps between two groups at 3^rd^ and 7^th^ postoperative day. On the 3^rd^ day, flap necrotic area didn’t show apparent difference between the two group. On the 7^th^ day, flap necrotic area of the COX-2 inhibitor group was larger than control group. **B**. On the 7^th^ day, the flap necrotic area ratio in the the selective COX-2 inhibitor group (66.65±2.81)% was significantly enlarged than control group(48.81±2.33)% (**P<0.01).

After one day, all flaps swelled to some extent, and the distal area III showed dark purple and tissue edema without obvious necrosis.On the third day, flaps of area II and III in the control and experimental group showed brown focal or patchy necrosis. On the seventh day, the necrotic parts described above tend to fuse, scab and harden. The boundaries between necrotic and surviving parts were stable. The survival portion were appeared pink-white, tender, normal in its texture, grew fine hair while and it bled when cut with a scalpel. the necrotic one became black, rigid, glabrous and did not bleed when cut (Figure 1A). 

### Percentage of Necrotic Area

7 days after operation, the results of flap necrosis percentage are presented in (Figure 1B): the mean flap necrotic areas percentage in study group（66.65±2.81,)% was significantly enlarged than that of the control group（48.81±2.33,)%（P <0.01,).

### Histology

**Figure 2:**
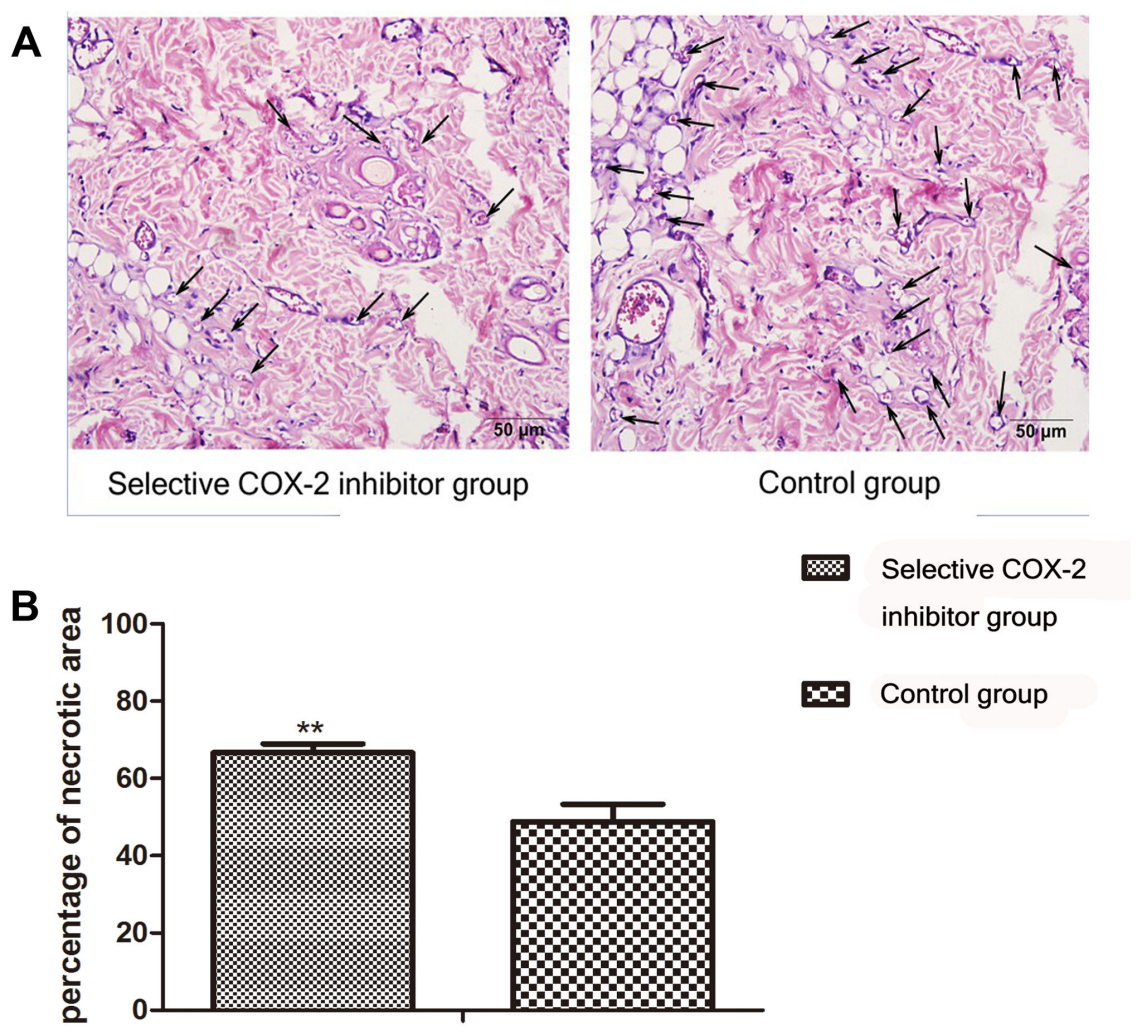
Comparison of angiogenesis in the intermediate area II between the two groups. **A**. Sections of the skin flap tissues from intermediate area II were stained with hematoxylin and eosin (HE). To evaluate the effect of COX-2 inhibitor on the neovascularization in the intermediate part of the flap, the number of vessels (arrows indicating vessels) was counted under original magnification, ×400. **B**. The number of vessels is indicated as vessel density per mm^2^. Mean vessel density in the selective COX-2 inhibitor group (15.4±4.4/mm^2^) was significantly lower than control group (27.2±4.1/mm^2^)(*P<0.05).

7 days after surgery ,the distal area of all flaps shared morphological similarity in histology. They all showed acute inflammatory infiltration. 90% of the meat film showed degeneration and necrosis of the muscle fibers. The respective neovascularization of I area in the selective COX-2 inhibitor group and saline group were 30.7±5.1/mm^2^ and 31.1±5.3/mm^2^. There was no statistically significant difference (P > 0.05 ). The respective neovascularization of II area were 15.4±4.4/mm^2^ vs 27.2±4.1/mm^2^ (Figure 2A). The difference was statistically significant (P < 0.05 ) (Figure 2B).

### Immunohistochemistry for COX-2 and VEGF

**Figure 3:**
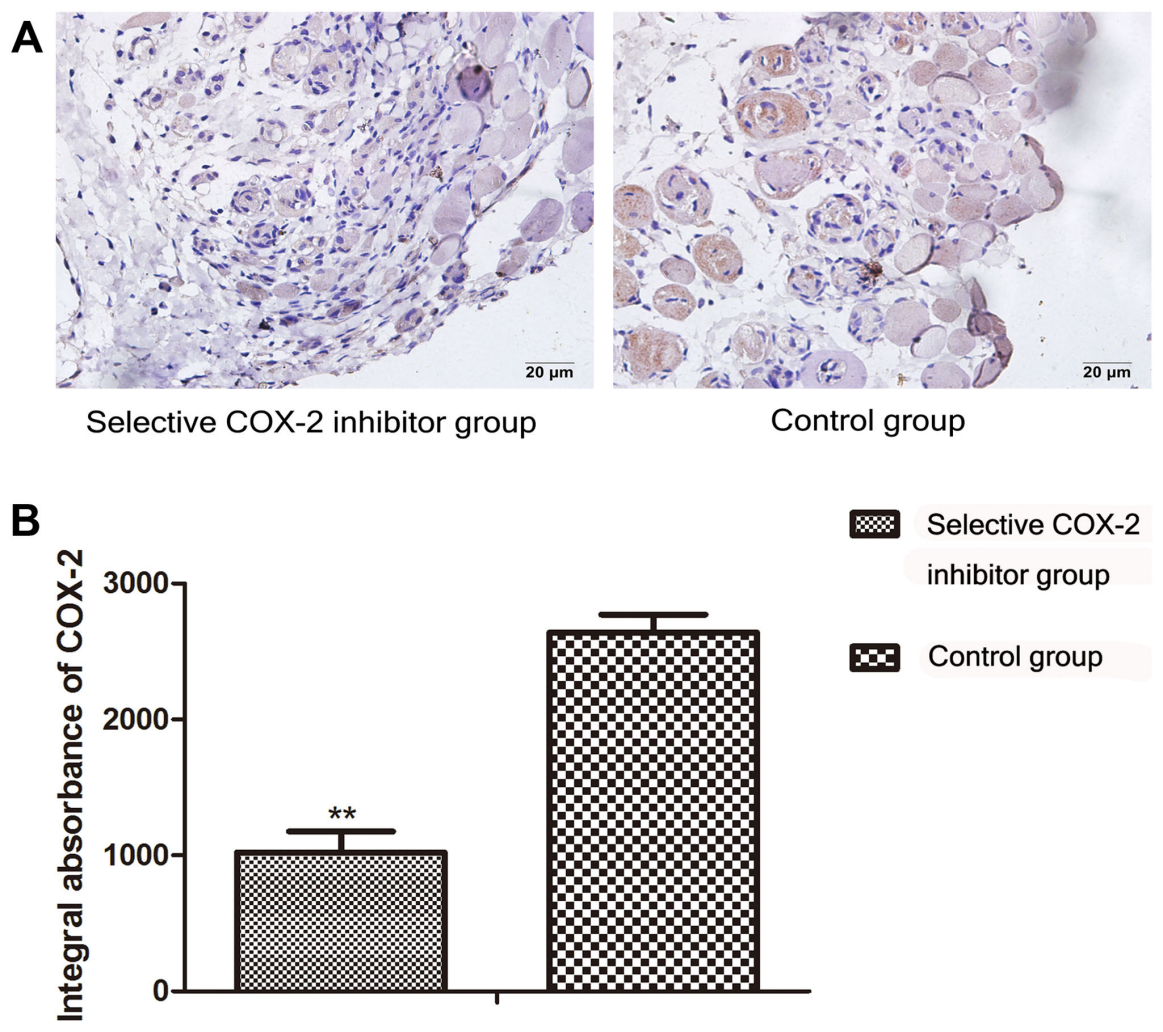
Comparison of COX-2 expression in the intermediate area II between COX-2 inhibitor and control group. **A**. To evaluate the expression of COX-2 protein in the intermediate area II in the two groups, sections of the tissues were taken for immunohistochemistry test and observed under original magnification, ×400. **B**. Integral absorbance (IA) value was detected to compare the level of COX-2. The IA of COX-2 in the selective COX-2 inhibitor group(1022.45±153.1) was lower than control group(2638.05±132.2) (**P<0.01).

**Figure 4:**
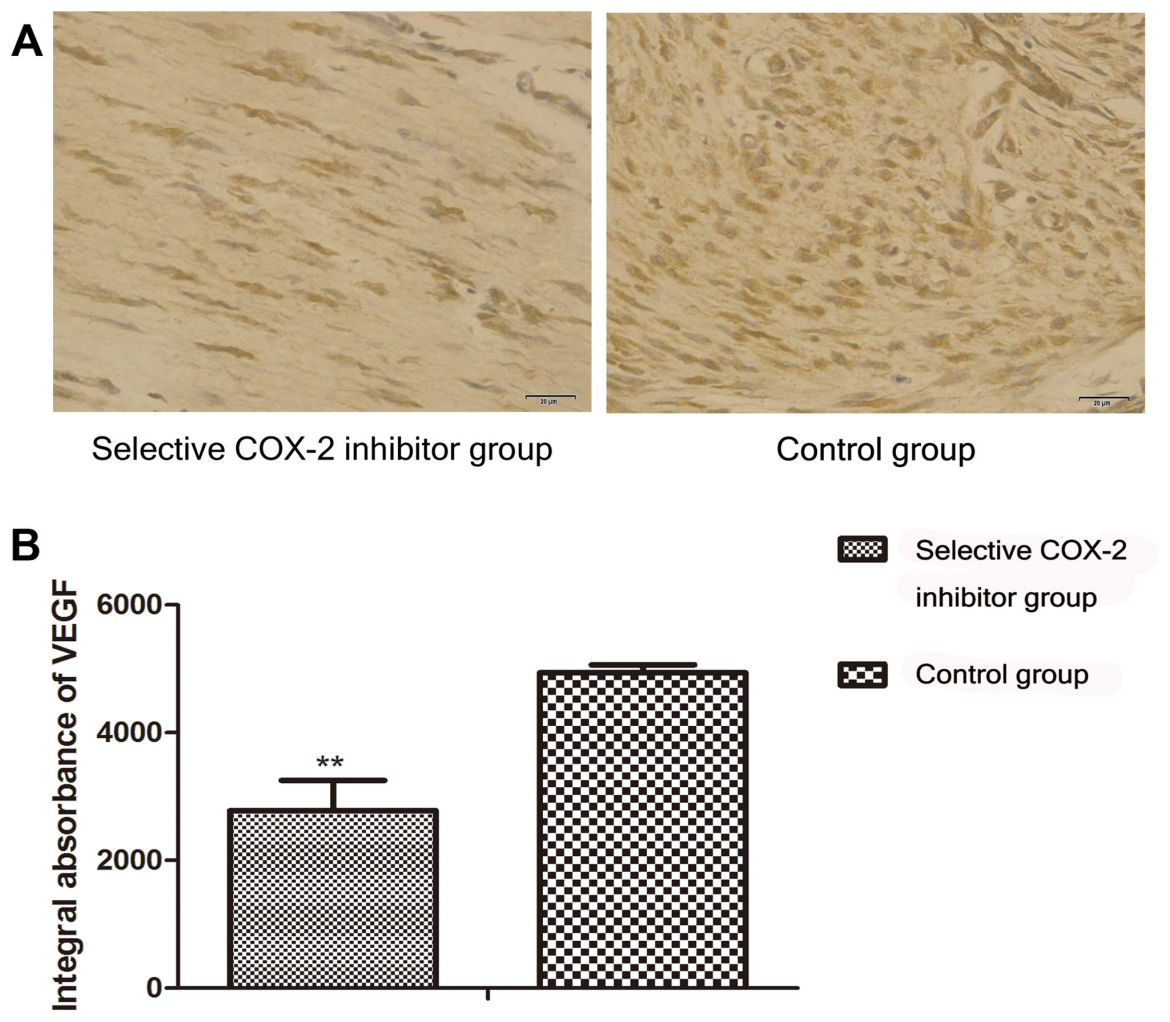
Comparison of VEGF expression in the intermediate area II between COX-2 inhibitor and control group. A. To evaluate the expression of VEGF protein in the intermediate area II in the two groups, sections of the tissues from II area were taken for immunohistochemistry test and observed under original magnification, ×400. **B**. Integral absorbance (IA) value was detected to compare the level of VEGF. The IA of VEGF in the selective COX-2 inhibitor group(2779.45±472.0) was lower than control group(4938.05±123.6) (**P<0.01).

At 7th day postoperatively, the results of immunohistochemical staining of the two groups were as follows. The IA of COX-2 in study group was 1022.45±153.1, and in control group was 2638.05±132.2 (Figure 3A). The difference was statistically significant (P < 0.01 ) (Figure 3B). The IA of VEGF of the two groups were 2779.45±472.0 vs 4938.05±123.6 respectively (Figure 4A). The difference was statistically significant（P <0.01,)(Figure 4B).

### Western Blot Assay for COX-2 and VEGF

**Figure 5:**
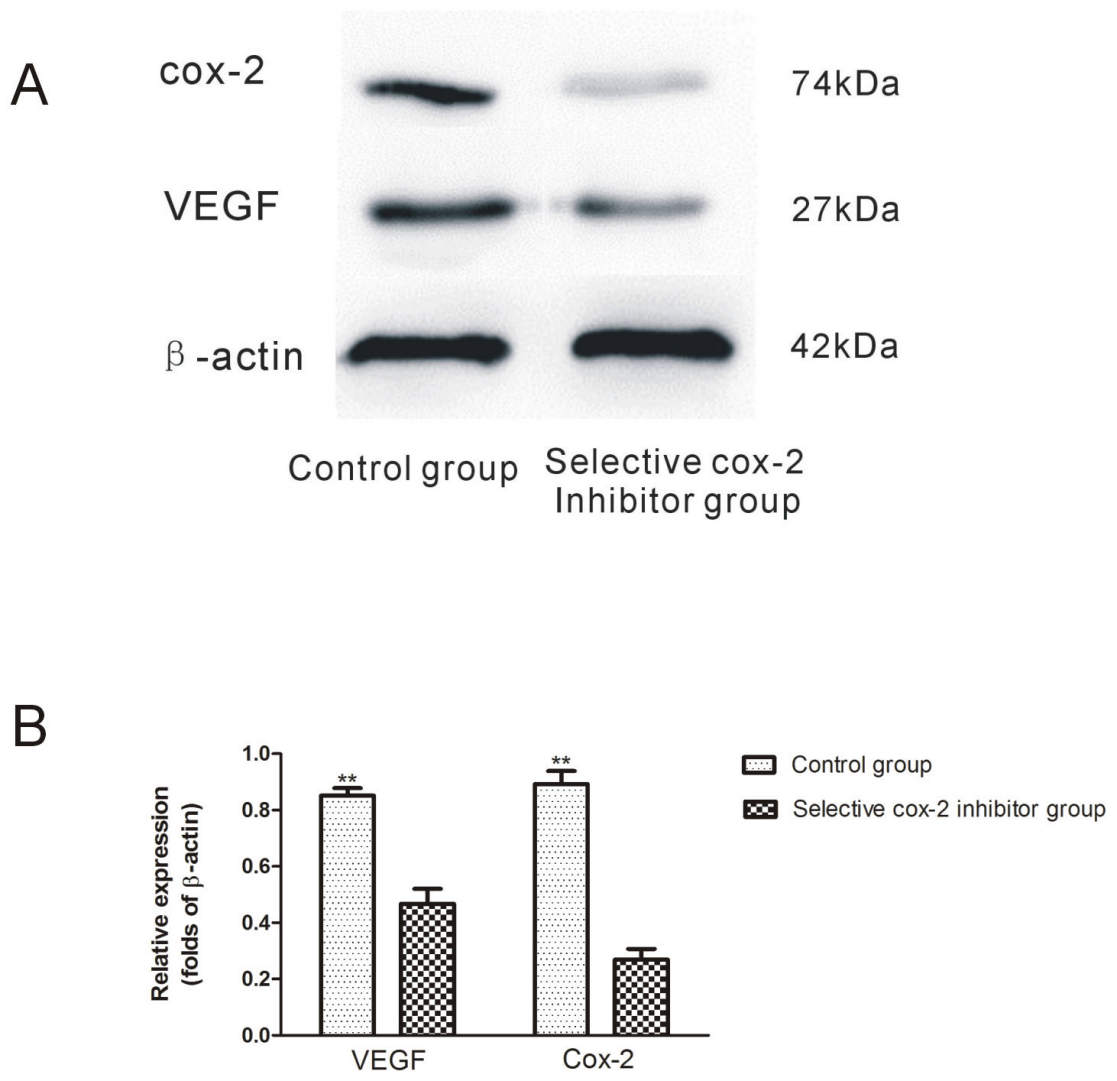
Western blot assay of COX-2 and VEGF protein expression in the intermediate area II flap tissue between COX-2 inhibitor and control group. **A**. Expression of VEGF, COX-2 by western blot. **B**. Densitometry results of VEGF and COX-2 protein expression between groups. Values are the mean ±SD( **P<0.01).

Western blot analysis confirmed the immunohistochemistry results. In the COX-2 inhibitor group, the expressions of COX-2 and VEGF protein were remarkably down-regulated as compared with the control group (Figure 5).

## Discussion

 Cyclooxygenase (COX) is the rate-limiting enzyme in the biosynthesis of prostaglandins. There are two main isoforms of COX identified so far, COX-2 is an early response gene and induced by many pro-inflammatory cytokines, including endotoxin, cytokines, mitogens and other stimuli, whereas COX-1 is constitutively expressed in most of the tissues [19]. Selective COX-2 inhibitors specifically inhibiting inducible COX-2 isoform than the constructive COX-1, are thought to provide better gastrointestinal tolerability and fewer gastrointestinal related side effects [1], and are extensively prescribed for the treatment of pain and inflammation associated with acute tissue damage due to injury or surgery. 

 Some documents indicated that neovascularization induced by exogenous VEGF seems to be the biological mechanism, which leads to the improvement of flap survival. VEGF is a potent endogenous stimulator of angiogenesis [20,21], a process which is believed to be essential for neovascularisation to occur, and increased vascular permeability [22,23]. In addition, VEGF can cause vasodilatation, partly through stimulation of nitric oxide synthase in endothelial cells, and can also stimulate cell migration and inhibit apoptosis [24]. It is expressed in developing blood vessels [25] and its receptors are found exclusively on endothelial cells [26,27]. When tissue is subjected to hypoxia or endothelial damage, expression of the VEGF protein is up-regulated [28]. Studies have confirmed that VEGF expression results in neovascularisation, increased flood flow and pressure, improved muscle function and measurable improvements in tissue viability [29]. 

 Evidences demonstrate that up-regulation of COX-2 correlates with VEGF expression [30] and COX-2-derived PGE_2_ can stimulate angiogenesis by induction of VEGF [31]. In our study, immunohistochemistry staining shows that COX-2 expression is significantly reduced in the treatment group comparing with the control group, and VEGF level was consistently deregulated in the treatment group. The density of new vessels in the histological analysis significantly decreased and the necrotic area of the flap enhanced after administration of selective COX-2 inhibitor. Consequently, the process of angiogenesis is suppressed, low distribution of new vessels in the impaired tissue result in inadequate oxygen supply and free radicals formation, which might be part of the mechanisms explaining the adverse effect of selective COX-2 inhibitors on wound healing. Our study demonstrated that selective COX-2 inhibitor would reduce VEGF synthesis and have adverse effect of on random skin flap survival. A clinical study have suggested that the use of selective COX-2 inhibitors was associated with an increased incidence of the failure of free vascular flaps [32], which is consistent with our findings. In addition, the histology analysis showed that there was no statistically significant difference of the respective neovascularization of I area in the selective COX-2 inhibitor group and control group, but the difference of the respective neovascularization of II area was statistically significant . We suppose the inconsistence would owing to that in the intermediate area II, the blood supply was limited while ischemia-reperfusion injury and inflammation was apparent, the production of COX-2 and PGs was massive in the tissue of control group but low level in the COX-2 inhibitor group owing to drug effect, so the level of VEGF was higher in the control group, which led to lesser neovascularization in COX-2 inhibitor group in II area. In the proximal area I, where the blood supply was enough while with little ischemia-reperfusion injury and inflammation, the production levels of COX-2 and PGs are low in each group, the expression of VEGF might has no significant difference between two groups, thus there was no statistically significant difference of respective neovascularization in I area.

 Studies explained that selective COX-2 inhibitors decrease the amount of prostacyclin (PGI2), a vasodilator, while having no effect on thromboxane A_2_, a potent vasoconstrictor and inducer of platelet aggregation. This disruption in the balance of these two substances might result in prothrombotic conditions [33], which would also attenuate wound healing in many tissues [11]. The COX-2 enzyme enables prostaglandin release and inflammatory response, Ott E et al. [12] demonstrated that the selective COX-2 inhibitors impede reparative inflammatory responses and were associated with a significantly higher incidence of wound infections. In addition, selective COX-2 inhibitors are reported to remarkably increase the risk of heart attacks [34–36], strokes and other cardiovascular problems [37,38].

 COX-2-selective NSAIDs are typically more tolerable than nonselective NSAIDs because they lack many of the side effects associated with COX-1 inhibition. These drugs are, therefore, widely prescribed for acute tissue damage due to injury or surgery, and often used chronically and at high doses [4]. Though COX-2-selective NSAIDs are effective as analgesics, the results of this study show that selective COX-2 inhibitors has adversely effects on random skin flap survival. We should see fairly clearly the side effect and reconsider the pain management on the patients of skin flap.
